# Plant growth promoting traits of selected psychrotolerant bacteria: a genomic basis of biocontrol, nutrient acquisition and stress tolerance

**DOI:** 10.1007/s11274-026-04994-y

**Published:** 2026-05-08

**Authors:** Ashira Roopnarain, Muyiwa Ajoke Akindolire, Thierry Alexandre Pellegrinetti, Haripriya Rama

**Affiliations:** 1 Microbiology and Environmental Biotechnology Research Group, Agricultural Research Council - Natural Resources and Engineering, Pretoria, South Africa; 2 Department of Environmental Sciences, College of Agriculture and Environmental Sciences, University of South Africa – Florida Campus, Private Bag X6, Florida, 1710 South Africa; 3 Département de phytologie, Faculté des sciences de l’agriculture et de l’alimentation, Université Laval, Québec City, Canada

**Keywords:** Abiotic stress, Biocontrol, Digestate, Plant growth promoting traits, Psychrotolerant bacteria, Whole genome sequencing

## Abstract

Psychrotolerant, plant growth promoting microorganisms represent valuable allies for sustainable agriculture in cold-stressed environments. This study employed whole-genome sequencing and comparative genomics to characterize three such psychrotolerant bacterial isolates possessing plant growth promoting traits. Average Nucleotide Identity (ANI), Type (Strain) Genome Server and phylogenomic reconstruction identified the isolates as *Comamonas jiangduensis*, *Pseudomonas rhodesiae*, and a putative novel *Acinetobacter* species. Functional profiling using PGPg_Finder revealed that all isolates possessed genes associated with plant growth promotion and abiotic stress tolerance, with *P. rhodesiae* boasting the most extensive repertoire of plant-beneficial genes. Pangenome analysis highlighted both shared and isolate-specific genetic features, providing insight into the functional differentiation among taxa. Moreover, the diverse biosynthetic gene clusters (BGCs) support the potential of these isolates in biocontrol, nutrient acquisition, and stress mitigation. RhizoSMASH analysis further indicated the genetic capacity for utilization of root exudates, suggesting rhizosphere compatibility despite the digestate origin of the isolates. Whilst predicted virulence determinants were detected in *P. rhodesiae*, it is postulated that these genes are related to ecological fitness and isolate resilience, rather than its pathogenicity; nevertheless, comprehensive biosafety assessment is required prior to application. Overall, the findings underscore the potential of all isolates for the enhancement of crop performance and highlight psychrophilic anaerobic digestate as an underexplored microbial reservoir. Notably, this study uniquely reports the first genome-enabled characterization of a putative novel cold-tolerant *Acinetobacter* species.

**Supplementary Information:**

The online version contains supplementary material available at 10.1007/s11274-026-04994-y.

## Introduction

Biotic and abiotic stresses as well as their concurrence, primarily induced by climate change, have affected global crop productivity (Pandey et al. 2017). Some of the major abiotic stresses include nutrient deficiency, extreme temperatures, salinity, acidity, heavy metal toxicity, flooding and drought, which retards crop development and leads to reduced yields. Similarly, biotic stresses, such as certain pathogenic microorganisms, weeds and insect pests, have detrimental impacts on crop productivity (Pandey et al. 2017). Conventional chemical fertilizers, herbicides, and pesticides have been predominantly applied to rapidly increase food production. However, the intensive application of such chemicals has led to environmental pollution as well as the gradual reduction in crop response (Zhao et al. 2024) and soil quality (Sharma et al. 2024).

To ensure food security and enhance crop productivity under stressed conditions, research and development on biofertilizer formulation and application as a substitute to chemical interventions has become inevitable. Biofertilizers comprise plant growth promoting (PGP) microorganisms that aid crop production, for instance, by conferring plant resilience to abiotic (Abd El-Daim et al. 2019) and biotic (Wang et al. 2023) stresses and improving nutrient acquisition for healthy plant development (Nabati et al. 2025). Some of the significant microbial traits that can aid in promoting plant resilience and growth include phosphate solubilization, nitrogen fixation, biofilm formation, siderophore and indole acetic acid (IAA) production (Akindolire et al. 2025), as well as production of antimicrobial compounds (Chen et al. 2025).

Although biofertilizers are promising solutions for climate-resilient and sustainable agriculture, several challenges preventing their success include negative interactions with the native microbiome, selection of microbes with ecologically irrelevant traits, as well as variability in soil properties and environmental conditions based on region (Mitter et al. 2021). Another key challenge includes the ineffective formulation of the biofertilizer (Fadiji et al. 2024). It is therefore important to consider native microorganisms with relevant and beneficial PGP traits for biofertilizer formulation. Potential microorganisms can be isolated from numerous, local sources such as rhizospheres (soil around roots) (Nabati et al. 2025), endospheres (interior plant tissue) (Raimi and Adeleke 2023), water bodies (Duong et al. 2021) and digestate arising from anaerobic digesters (Akindolire et al. 2025), followed by screening for relevant PGP traits.

Typically, screening only involved culture-dependent approaches to assess the phenotypic PGP traits of microbial isolates (Mitter et al. 2021). However, over recent years and with the advancement of sequencing technology and bioinformatics tools, omics interventions, such as whole-genome and metagenomic sequencing, have been adopted during screening for sophisticated identification, trait comparison and selection of promising PGP isolates (Pellegrinetti et al. 2024). Integrating omics approaches facilitates a deeper understanding of the potential PGP capabilities, plant-microbe interactions as well as their holistic regional and ecological relevance for application (Sahoo et al. 2025). As such, this approach enables effective formulation of biofertilizers comprising omics-guided microbial consortia with greater potential for efficacy and ability to persist for the intended application.

In a recent study, a PGPg_finder pipeline was developed allowing for the holistic identification and comparison of a plethora of curated genes specifically relating to plant growth promotion from whole-genome and metagenomics data (Pellegrinetti et al. 2024). In addition to genes associated with PGP traits, tools like antiSMASH (antibiotics and secondary metabolite analysis shell) have been developed to mine microbial genomes for biosynthetic gene clusters (BGCs), and their potential to synthesize secondary metabolites and antimicrobial compounds for biocontrol applications (Blin et al. 2023). Furthermore, genomes can be screened for potential pathogenic and virulence traits to guide further phenotypic evaluation of isolates prior to downstream application to prevent exposure to hazardous biological agents (Zhang et al. 2021). Utilization of such interventions during microbial screening can pointedly advance agricultural research toward sustainable crop production using biofertilizers.

Thus far, a diverse range of microorganisms have been investigated for genomic-based and phenotypic PGP traits (Rikame and Borde 2022; Kumar et al. 2022; Patakova et al. 2024; Sharma et al. 2024; Sun et al. 2025; Chen et al. 2025). Nonetheless, a massive number of microorganisms with significant potential in the biofertilizer industry remain unidentified (Singh and Kumar 2024). Psychrotolerant bacteria, previously isolated from digestate arising from a cold-acclimated anaerobic digester fed with cattle manure, were screened using culture dependent approaches and were shown to have several PGP traits (Akindolire et al. 2025). This study aimed to further screen and unravel the whole genomes of the three most promising psychrotolerant bacteria (*Comamonas jiangduensis*,* Pseudomonas rhodesiae* and *Acinetobacter* sp.), isolated in the study conducted by Akindolire et al. (2025), for potential application as a biofertilizer. In particular, whole genome sequence data was used to identify the isolates and screen for comprehensive plant growth promoting traits, biosynthetic potential, and pathogenicity. In essence, this study provides a comprehensive genome-resolved assessment of three psychrotolerant bacteria with plant growth-promoting traits from a previously underexplored digestate environment, linking taxonomic identity, functional potential, and ecological adaptation.

## Materials and methods

### Bacterial isolation from psychrophilic digestate

The bacterial isolates used in this study were obtained from psychrophilic anaerobic digestate generated during a batch acclimation experiment conducted at the Agricultural Research Council–Natural Resource and Engineering in Pretoria, South Africa (25° 44′ 19.4″ S, 28° 12′ 26.4″ E). Anaerobic digestion was gradually acclimated from mesophilic (30 °C) to psychrophilic conditions (15 °C) over a 42-day period, and isolation was performed at 15 °C, reflecting cold-stress conditions representative of winter operation in temperate and subtropical regions (Akindolire et al. 2025). Briefly, 1 g of digestate was diluted in 9 ml of sterile distilled water. Serial dilution followed up to 10^− 8^ and 100 µl aliquots of each dilution was spread on Nutrient Agar (Central Drug House (P) Ltd., New Delhi, India) plates and incubated at 15 °C for 48 h. After incubation, pure isolates were obtained by sub-culturing morphologically distinct bacterial colonies. Pure isolates were preserved in 20% glycerol at − 80 °C. Isolate31, isolate33, and isolate55 were selected from a larger pool of psychrotolerant bacterial isolates reported in a previous study, in which the corresponding strains (A3-1, A3-3, and B5-5, respectively) were subjected to phenotypic characterization (Akindolire et al. 2025). These three isolates were chosen based on their superior plant growth-promoting traits at 15 °C, including multiple nutrient acquisition and colonization-related traits, as reported by Akindolire et al. (2025). In addition, the isolates were chosen to represent different bacterial genera, enabling comparison of how distinct psychrotolerant taxa, from the same digestate environment, differ in their plant growth-promoting potential, biosynthetic capacity, and predicted biosafety profiles.

### DNA extraction and whole genome sequencing

The quick-DNA fungal/bacterial kit D6005 (Zymo Research, United States) was used for Genomic DNA extraction from isolate31, isolate33 and isolate55. The quantity and quality of the extracted DNA was assessed using a Qubit 2.0 fluorometer (Invitrogen, California, United States) and 1% agarose gel electrophoresis, respectively. The DNA samples were sent to the Agricultural Research Council-Biotechnology Platform for whole genome sequencing. Briefly, sequencing libraries were generated using the MGIEasy Universal DNA Library Prep Set (MGI Tech) following manufacturer’s instructions and sequenced on the MGI DNBSEQ‑G400 platform using a 150 bp paired-end sequencing strategy.

### Whole genome sequence analysis

The whole genomes of isolate31, isolate33 and isolate55 were analyzed on the DOE Systems Biology Knowledgebase platform (Kbase, http://kbase.us/) (Arkin et al. 2018). Briefly, raw sequence reads were subjected to quality control using FastQC (v0.12.1) (Andrews 2010), followed by quality and adapter trimming using Trimmomatic (v0.36) (Bolger et al. 2014). Cleaned reads were assembled using SPAdes Genome Assembler software (v3.15.3) (Bankevich et al. 2012; Prjibelski et al. 2020), then genome completeness and level of contamination were evaluated with CheckM (v1.0.18) (Parks et al. 2015). Annotation of the isolate genomes followed using DRAM (Distilled and Refined Annotation of Metabolism) software (v0.1.2) (Shaffer et al. 2020), and taxonomic assignment was performed using GTDB-Tk (v1.7.0) (Chaumeil et al. 2020). Species-level delineation was conducted using Average Nucleotide Identity (ANI) comparisons with FastANI (v0.1.3) (Jain et al. 2018). Results related to genome completeness and contamination (CheckM), genome-based taxonomic assignment (GTDB-Tk), and metabolic potential inferred by DRAM are summarized in Supplementary Table S1.

### Genome mining

Outputs from KBase were used to further interrogate the isolate genomes with respect to isolate identification, plant growth promotion potential, assessment of virulence determinants as well as visualization of assemblies.

#### Isolate identification and comparative pangenome analysis

Genome assemblies were submitted to the Type (Strain) Genome Server (TYGS) webserver (https://tygs.dsmz.de) for initial taxonomic placement using digital DNA-DNA hybridization (dDDH) and phylogenomic analysis using the GBDP algorithm and FastME-based tree construction (Meier-Kolthoff and Göker 2019). Phylogenomic relationships were thereafter inferred, using genomes of related strains identified through TYGS, and reconstructed using the GToTree pipeline (Lee 2019). This workflow identifies and aligns conserved bacterial marker genes to generate a concatenated alignment suitable for genome scale phylogenetic inference. The analysis was performed using the bacterial marker gene set specified with the parameter “-H Bacteria”, and *Synechococcus elongatus* PCC 6301 was used as an outgroup to properly root the tree. The resulting phylogenetic tree was visualized and annotated using the Interactive Tree of Life (iTOL) platform (Letunic and Bork 2021).

A comparative pangenome analysis was performed to assess shared and isolate-specific/unique genetic features among the studied strains and closely related reference genomes. This analysis was performed using anvi’o (Eren et al. 2015). Separate pangenome analyses were conducted for each isolate together with their respective TYGS related reference strains. This approach allowed characterization of the core genome, representing conserved genes shared across strains, and the accessory genome, representing strain specific genes associated with functional diversification and adaptation.

#### Plant growth promoting gene prediction and visualization

The genome workflow (genome_wf) on the PGPg_finder pipeline (https://github.com/tpellegrinetti/PGPg_finder) was used to predict plant growth promoting genes from uploaded isolate genome files (Pellegrinetti et al. 2024). Briefly, the pipeline employed Prodigal v2.6.3 for the first step, i.e. gene prediction (Hyatt et al. 2010). Thereafter, sequence annotation was conducted using the alignment tool, DIAMOND (v2.1.8.162) (Buchfink et al. 2015) that facilitated alignment against the PLaBAse – PGPT- database (https://plabase.cs.uni-tuebingen.de/pb/download.php) (Patz et al. 2021). The results were visualized as heatmaps and a Venn diagram to highlight unique PGP traits and functional overlaps between the three isolates. All graphs were constructed and customized in R using the ggplot2, pheatmap, and VennDiagram packages (Wickham 2011; Chen and Boutros 2011; Kolde 2015).

#### Biosynthetic and root-associated gene cluster analysis

AntiSMASH v7.0 was used to identify, annotate, and analyze secondary metabolite biosynthetic gene clusters (BGCs) in all three isolates using default parameters (Medema et al. 2011). Proksee was used to integrate the resulting BGCs into circular chromosome maps of the respective isolates (https://proksee.ca/) (Grant et al. 2023). Prediction of root-associated catabolic gene clusters (rCGCs) associated with rhizosphere competence was performed using RhizoSMASH (Crits-Christoph et al. 2018) with default parameters. The analysis was conducted using GenBank annotation files generated with Prokka (Seemann 2014). KnownClusterBlast comparisons were included to identify homologous biosynthetic gene clusters and assess potential functional similarity to characterized clusters.

#### Resistance and virulence gene detection

ABRicate (v1.0.1) was used to assess the presence of virulence-associated and antimicrobial resistance genes in the respective isolate genomes (Seemann 2016). Three databases were used for screening, including the Virulence Factor Database (VFDB) (Chen et al. 2016), the Comprehensive Antibiotic Resistance Database (CARD) (Alcock et al. 2023), and the NCBI Bacterial Antimicrobial Resistance Reference Gene Database (Feldgarden et al. 2021). Hits were considered significant when identity and coverage exceeded 90%.

## Results and discussion

### Genome assembly, taxonomic classification and comparative analysis

A summary of the genome assembly characteristics and taxonomic assignment of the three isolates is presented in Table 1. Comparatively, isolate55 exhibited the largest genome size (5.77 Mb) and most predicted genes (5,133). The genomic features of isolate55 indicate a highly complex architectural organization, suggesting an expanded functional repertoire relative to the other isolates. The smaller genome sizes and lower predicted gene counts of isolate31 and isolate33 could be linked to their taxonomy, reduced metabolic potential and/or narrower ecological specialization (Rodríguez-Gijón et al. 2023). For all isolates, assembly quality matrices including number of contigs and N50 (length of the shortest contig that accumulatively show 50% or more of the genome size) were assessed. All genomes contained less than 100 contigs and a N50 of > 5000 (Table 1), implying good assembly quality and congruity and supporting downstream functional annotation, gene prediction, and BGC identification (Riesco and Trujillo 2024).

**Table 1:** Genome assembly characteristics and taxonomic assignment results for isolates obtained from a cold-adapted anaerobic digester

Characteristic	Isolate31	Isolate33	Isolate55
Genome size (bp)	3,388,837	3,053,062	5,765,167
G + C ratio (%)	58	43	60
No. of contigs	30	29	21
N50	274,459	255,348	746,930
No. of predicted genes	2942	1875	5133
Average nucleotide identity (%)	95.93	Genome not assigned to closest species as it falls outside its pre-defined ANI radius	97.76
Closest genome	*Comamonas jiangduensis*	*Acinetobacter* sp.	*Pseudomonas rhodesiae*

The ANI values supported the assignment of isolate31 as *Comamonas jiangduensis* (95.93%) and isolate55 as *Pseudomonas rhodesiae* (97.76%). However, genome-based taxonomic assignment using the GTDB-Tk framework was unable to assign isolate33 beyond the genus level of *Acinetobacter*. Notably, GTDB-Tk reported that isolate33 fell outside the predefined species ANI radius of its closest reference genome, indicating that its highest ANI value was below the accepted species delineation threshold of 95%. This suggests that isolate33 may represent a previously undescribed *Acinetobacter* species, indicating its putative novelty (Chun et al. 2018). The taxonomic classification using ANI was corroborated by the phylogenomic findings using the TYGS webserver where isolate31 and isolate55 exceeded the species dDDH threshold of 70%, thereby confirming species-level assignment (Chun et al. 2018). In essence, isolate31 was identified as *C. jiangduensis* (dDDH = 71.1%) and isolate55 was confirmed as *P. rhodesiae* (dDDH = 81.3%). Isolate33 showed a maximum dDDH of 57.8% with *Acinetobacter iwoffii*, indicating it is a putative novel species within the genus *Acinetobacter* (Chun et al. 2018; Meier-Kolthoff and Göker 2019). The isolates will henceforth be referred to by their closest genome names (Table 1).

**Fig. 1:**
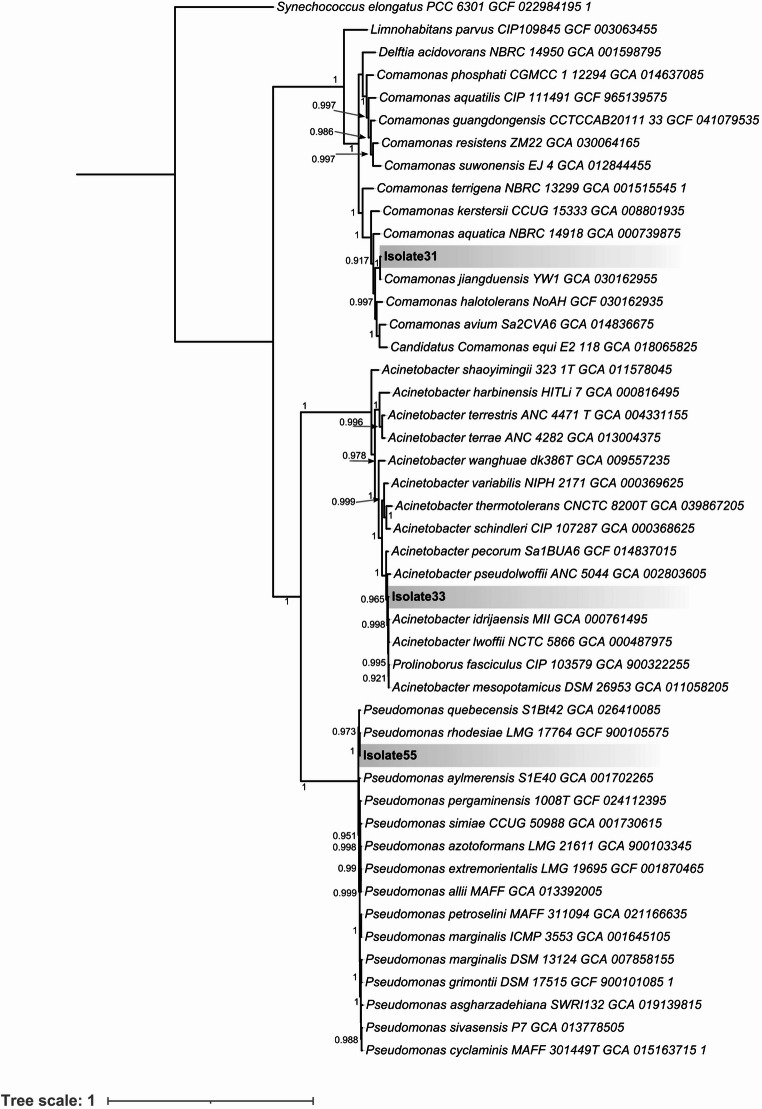
Genome-based phylogenomic tree reconstructed using the GToTree pipeline. Closely related reference genomes identified via TYGS were included, with *Synechococcus elongatus* PCC 6301 used as an outgroup. Node support values are shown at branch points. Isolate31 and isolate55 cluster with *Comamonas jiangduensis* and *Pseudomonas rhodesiae*, respectively, while isolate33 forms a distinct lineage within *Acinetobacter*, consistent with ANI-based classification

To obtain higher-resolution insight into the evolutionary relationships of the isolates, the GToTree pipeline was utilized to construct a genome-based phylogenomic tree (Fig. 1) which revealed clear taxonomic placement for the isolates. Isolate31 clustered with *Comamonas jiangduensis* YW1, supporting its classification within the genus *Comamonas*. In contrast, isolate33 did not cluster tightly with any single reference strain but was positioned near *Acinetobacter idrijaensis* MIII and *Acinetobacter pseudolwoffii* ANC 5044, suggesting phylogenetic proximity within the *Acinetobacter* lineage while potentially representing a distinct genomic variant. Isolate55 formed a clade with *Pseudomonas rhodesiae* LMG 17,764, confirming its affiliation within the genus *Pseudomonas*. These results provide robust phylogenomic evidence for the taxonomic positioning of the three isolates. 

In addition to genome assembly and taxonomic classification, a comparative pangenome analysis was performed to assess shared and isolate-specific genetic features among the studied strains and closely related reference genomes (Fig. S1). The pangenome analysis revealed distinct levels of genomic conservation and diversification among isolates. Isolate31 presented 1,132 core genes and 200 accessory genes, indicating moderate genomic variability relative to related strains. Isolate33 showed a larger conserved gene fraction, with 1,697 core genes and only 93 accessory genes, suggesting greater genomic conservation. In contrast, Isolate55 exhibited 2,287 core genes and 219 accessory genes, representing the largest accessory genome among the isolates and indicating higher genomic plasticity. Accessory genes may contribute to strain specific metabolic capabilities and ecological adaptation. Importantly, this genomic framework provided the basis for downstream genome-resolved analyses conducted in this study, including functional annotation and comparative assessment of plant growth-promoting potential, by contextualizing shared and isolate-specific genetic capacity. 

### Functional annotation using PGPg_finder

**Fig. 2:**
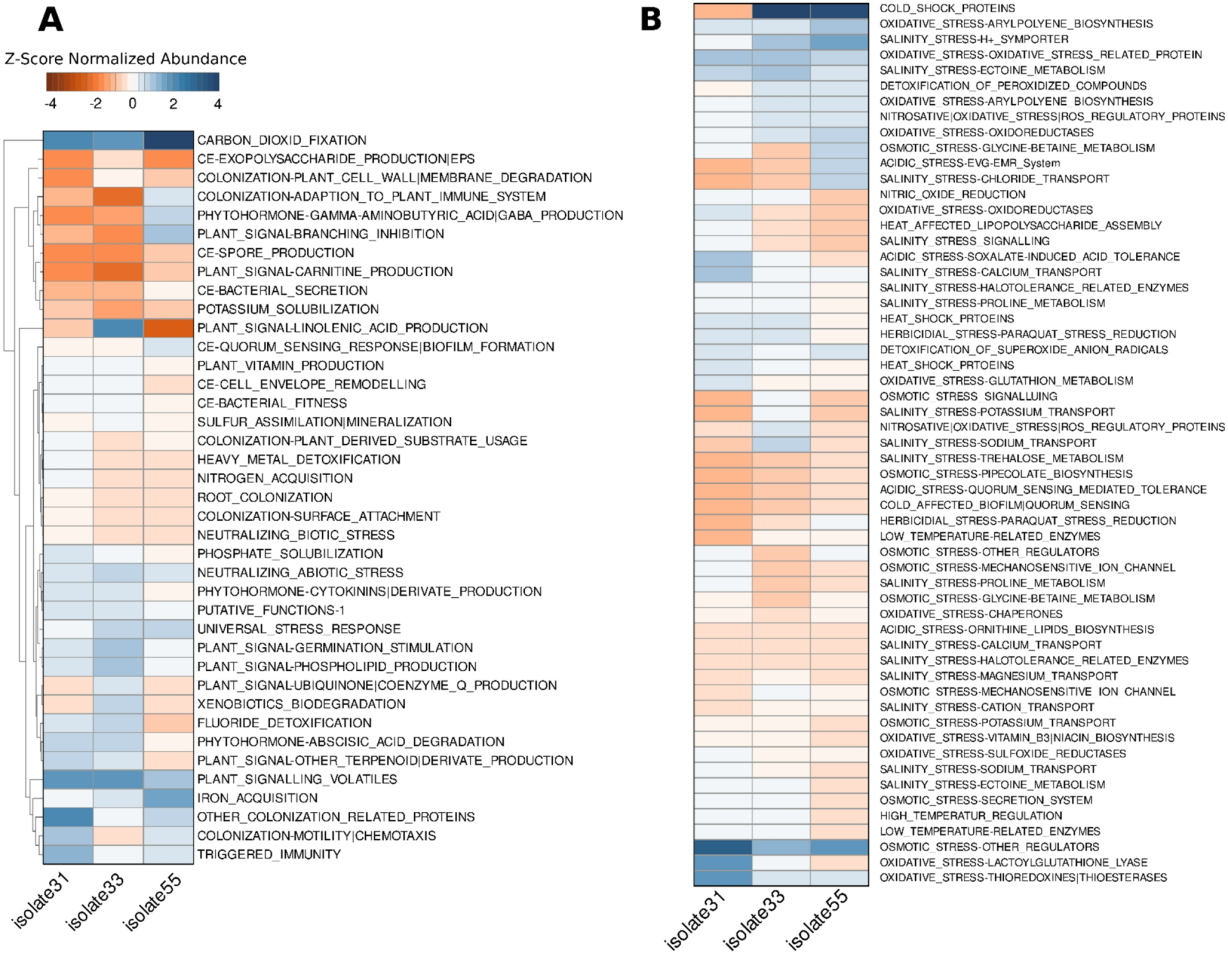
PGPg_Finder-derived functional profiles of plant growth-promoting (PGP)-associated genes identified in isolate31, isolate33, and isolate55 (*Comamonas jiangduensis*, *Acinetobacter* sp., and *Pseudomonas rhodesiae*, respectively). (**A**) Heatmap showing the distribution of PGP-associated functional categories related to nutrient acquisition, phytohormone-associated functions, iron acquisition, and colonization/motility for each isolate. (**B**) Heatmap showing the distribution of functional categories associated with stress response and detoxification, including genes linked to tolerance of abiotic stress conditions

**Fig. 3:**
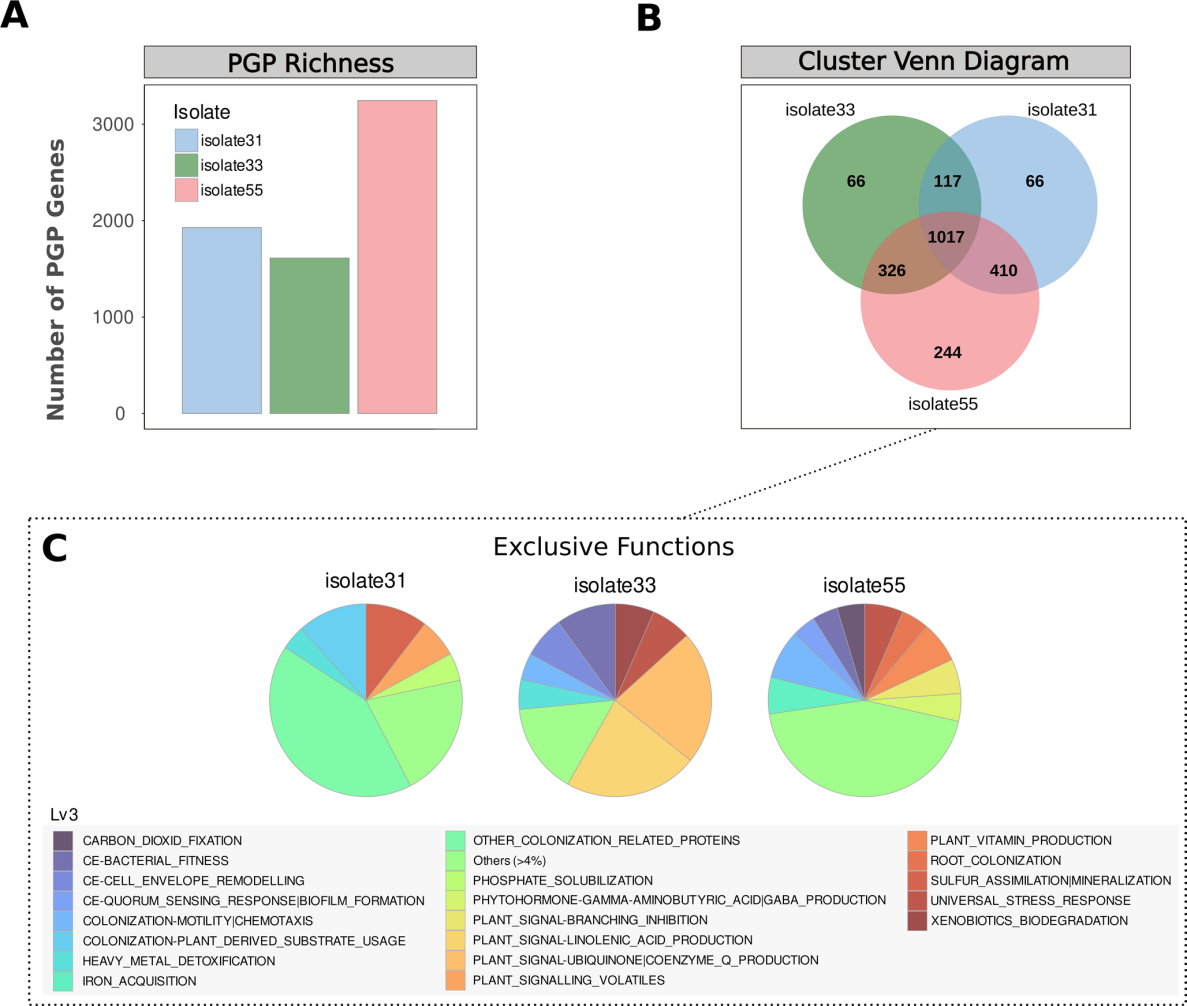
Comparative distribution and uniqueness of plant growth-promoting (PGP) genes across isolate31, isolate33, and isolate55 (*Comamonas jiangduensis*, *Acinetobacter* sp., and *Pseudomonas rhodesiae*, respectively), based on PGPg_Finder output. (**A**) PGP gene richness expressed as absolute gene counts for each isolate, providing a comparative indicator of genomic capacity rather than gene expression or activity. (**B**) Venn diagram illustrating shared and isolate-specific PGP functional categories. (**C**) Functional categories uniquely represented in each isolate.

PGPg_Finder was used to assess the PGP potential of the isolates, *C. jiangduensis*, *Acinetobacter* sp. and *P. rhodesiae*. Distinct yet complementary strengths in relation to plant growth promotion across all isolates were observed (Fig. 2A). Whilst *P. rhodesiae* exhibited the widest spectrum of PGP genes, all isolates contained genes synonymous with plant growth promotion including nitrogen acquisition, phytohormone production, iron acquisition and phosphate solubilization genes, amongst others (Figs. 2A and 3A). Moreover, the isolates potential to be utilized as plant growth promoters is enhanced by their array of abiotic stress tolerance genes (Fig. 2B). A rich repertoire of abiotic stress tolerance genes was evidenced in all genomes with *P. rhodesiae* once again standing out as the most prolific in terms of stress tolerance. The isolates portrayed the genetic potential to withstand cold, heat, oxidative, salinity and osmotic stress, amongst others (Fig. 2B), underscoring their wide resilience particularly when employed in stressful soil environments.

Whilst *P. rhodesiae* contained the most extensive repertoire of PGP and stress tolerance genes, the elevated representation of phosphate-solubilization and heavy-metal detoxification genes in *C. jiangduensis* highlights a more specialized but ecologically valuable functional profile, particularly suited to environments where nutrient limitation and metal toxicity are prevailing constraints. Similarly, *Acinetobacter* sp., with its fewer PGP genes, still demonstrated elevated genes related to root colonization, several abiotic stress tolerance genes and plant signaling genes such as linolenic acid production, which implies potential as a biocontrol agent (Eng et al. 2021).

The functional profiles of all isolates identified in this study align with previously reported plant-beneficial traits in closely related taxa. For *C. jiangduensis* (isolate31), Fig. 2A revealed an enrichment of functional categories related to nutrient acquisition and colonization-motility. Whilst direct evidence demonstrating these PGP traits was not identified in literature, the species has been reported as a biosurfactant producer (Sun et al. 2013). Mechanistic reports further confirm that biosurfactant producing bacteria can contribute to nutrient mobilization and biocontrol in plant-associated bacteria (Sachdev and Cameotra 2013). For *Acinetobacter* sp. (isolate33), Fig. 2 indicates the presence of nutrient and iron acquisition as well as stress response and detoxification categories. These profiles are consistent with several published studies reporting plant growth promotion and abiotic stress tolerance by *Acinetobacter* strains through nutrient acquisition strategies and antioxidant activity (Mujumdar et al. 2023; Ali et al. 2023). For *P. rhodesiae* (isolate55), the vast representation of nutrient acquisition categories in Fig. 2, together with stress response functions aligns with reports of plant growth promotion and stress tolerance induced by *P. rhodesiae* in plant systems (Kang et al. 2007; Rolón-Cárdenas et al. 2021). Collectively, these comparisons indicate that the genomic profiles, and associated inferred PGP potential, identified in the isolates evaluated in the present study are consistent with PGP strategies previously reported for closely related taxa.

In addition to plant growth-promoting traits, all isolates demonstrated genomic features associated with psychrotolerance. The isolates were previously shown to grow and exhibit PGP traits, including nutrient mobilization, hydrolytic enzyme production, motility, and biofilm formation, at 15 °C (Akindolire et al. 2025). The present analysis provides genotypic insight into these previously reported phenotypic traits. Specifically, genes encoding cold-shock proteins, membrane adaptation systems, and compatible solute biosynthesis pathways were identified across all genomes. The co-occurrence of cold-tolerance genes with nutrient acquisition, root colonization, and stress mitigation genes emphasizes the ecological relevance of these isolates for plant growth promotion in cold-prone or seasonally fluctuating environments, where mesophilic biofertilizer strains may not be as effective.

Whilst the isolates demonstrated several shared gene clusters as evidenced in the Venn diagram (Fig. 3B) it is important to note that all isolates also possess exclusive genes (Fig. 3C). This speaks to the potential of co-culturing the isolates to convey synergistic benefits due to the presence of complementary genomic traits. However, the multi-isolate combinations must be evaluated in vitro and in planta to ensure that antagonism/competition between the isolates is reduced and maximal benefits are achieved (Wang et al. 2024).

To further contextualize the PGP-associated functional profiles of the studied isolates, a comparative PGPg_Finder analysis including closely related strains was performed (Fig. S2). The comparative PGPg_Finder analysis revealed that the studied isolates share core plant growth-promoting (PGP) functional categories with closely related strains, indicating a conserved genetic basis for plant-associated lifestyles. No uniquely enriched or highly distinctive gene sets were identified that strongly differentiated individual isolates. These findings suggest that plant associated functional traits are broadly distributed among related taxa rather than restricted to specific isolates. 

### Biosynthetic and root-associated gene cluster analysis

A diverse array of BGCs were evidenced in all three isolates (Fig. S3; Table 2). These BGCs consist of genes that encode enzymes and pathway regulators responsible for secondary metabolite synthesis. Secondary metabolites are natural microbial products that provide the microbe with several ecological advantages, as they facilitate competition, pathogenesis and symbiosis (Hibbing et al. 2010; Chen et al. 2019; Zhang et al. 2024). The specialized secondary metabolites encoded by BGCs are of particular importance in biofertilizer strains as they could underpin the strains effectiveness as an inoculant, due to its associated role in facilitating root colonization, plant growth promotion and pathogen antagonism, amongst other traits (Douka et al. 2025). An overview of the potential of the BGCs identified in *C. jiangduensis*,* Acinetobacter* sp. and *P. rhodesiae*, as it relates to biocontrol, nutrient acquisition and abiotic stress tolerance traits, is highlighted in Table 2. Notably, the most prolific isolate was identified as *P. rhodesiae*, which demonstrated several BGCs with an array of tentative functions that span biocontrol, nutrient acquisition and abiotic stress tolerance. However, the collective traits, if the isolates are used as a consortium, may provide the greatest benefits, as long as they do not compete with each other. The genomic potential, as gauged by the array of BGCs, further supports the ecological versatility and potential broad functionality of these cold-adapted isolates in agricultural applications.

**Table 2:** Biosynthetic gene clusters (BGCs) identified by antiSMASH and their predicted functions across *Comamonas jiangduensis*,* Acinetobacter* sp. and *Pseudomonas rhodesiae*

Biosynthetic gene cluster	Tentative function	Biocontrol	Nutrient acquisition	Abiotic stress tolerance	Reference
*Comamonas jiangduensis* (Isolate31)					
Terpene/ terpene-precursor	Produce volatile and antimicrobial metabolites with diverse ecological functions including pathogen suppression/defense, plant signaling and environmental stress protection.	✔	✖	✔	Yamada et al. (2015)
*Acinetobacter* sp. (Isolate33)					
Betalactone	Produce compounds with antimicrobial or cytotoxic activity.	✔	✖	✖	Robinson et al. (2019)
Arylpolyene	Produce pigments that function as antioxidants, protecting microbes from oxidative stress.	✖	✖	✔	Cimermancic et al. (2014)
RiPP-like	Produce peptides with antimicrobial roles relevant to pathogen suppression.	✔	✖	✖	Arnison et al. (2013)
Terpene-precursor	Produce volatile and antimicrobial metabolites with diverse ecological functions including pathogen suppression/defense, plant signaling and environmental stress protection.	✔	✖	✔	Yamada et al. (2015)
*Pseudomonas rhodesiae* (Isolate55)					
NRPS/NRPS-like	Produce nonribosomal peptide synthetases (NRPS) that synthesize small bioactive metabolites such as siderophores, antimicrobials and toxins that suppresses pathogens and facilitates nutrient acquisition.	✔	✔	✖	Miethke and Marahiel (2007); Ongena and Jacques (2008)
NAGGN	Encodes small amide metabolites that potentially supports microbial abiotic stress tolerance e.g. osmotic stress.	✖	✖	✔	Sagot et al. (2010)
Betalactone	Produce compounds with antimicrobial or cytotoxic activity.	✔	✖	✖	Robinson et al. (2019)
RiPP-like	Produce RiPPs with antimicrobial roles relevant to pathogen suppression.	✔	✖	✖	Arnison et al. (2013)
NRP-metallophore	Hybrid clusters producing metal-chelating compounds (metallophores), often linked to iron acquisition and microbial competition.	✔	✔	✖	Miethke and Marahiel (2007); Medema et al. (2015); Blin et al. (2021)
Redox-cofactor	Encodes redox-active cofactors that support microbial fitness by mitigating oxidative damage and enhancing energy metabolism. May also contribute to plant growth promotion e.g. through phosphate solubilization.	✖	✔	✔	Choi et al. (2008); Bashiri et al. (2019)
Terpene-precursor	Produce volatile and antimicrobial metabolites with diverse ecological functions including pathogen suppression/defense, plant signaling and environmental stress protection.	✔	✖	✔	Yamada et al. (2015)
Arylpolyene	Produce pigments that function as antioxidants, protecting microbes from oxidative stress.	✖	✖	✔	Cimermancic et al. (2014)

In addition to BGCs associated with secondary metabolism, gene clusters linked to rhizosphere-associated catabolic functions was also investigated using RhizoSMASH. The analysis revealed significant biosynthetic potential in all isolates. *C. jiangduensis* presented seven predicted biosynthetic gene clusters, including clusters associated with organic acid production. *Acinetobacter *sp. presented twelve predicted biosynthetic gene clusters, including pathways related to organic acids, phytohormones, and amino acid derived compounds. Whereas, *P. rhodesiae* showed the highest biosynthetic diversity, with nineteen predicted gene clusters, including pathways associated with aromatic compounds, organic acids, carbohydrates, and amino acid derived metabolites. These results indicate substantial metabolic potential and suggest possible roles in plant interaction, nutrient cycling, and ecological adaptation. Together, BGCs and root-associated catabolic gene clusters indicate complementary genetic potential for secondary metabolism and rhizosphere adaptation, supporting further evaluation of these digestate-derived isolates for plant-associated applications.

### Potential pathogenicity

#### *Comamonas jiangduensis* and *Acinetobacter* sp

It is imperative that the potential pathogenicity of any isolate used for biofertilizer development is ascertained to ensure safe and sustainable agricultural application as well as regulatory compliance (Vassileva et al. 2022). As such, ABRicate was used to establish the potential virulence and resistance gene profile of *C. jiangduensis*,* Acinetobacter* sp. and *P. rhodesiae* (Table S2). The absence of virulence genes in *C. jiangduensis* and *Acinetobacter* sp., supports their potential safety for agricultural use. However, it is important to note the presence of antibiotic resistance genes in both isolates. *C. jiangduensis* harbours putative resistance determinants to aminoglycosides whereas the putative novel *Acinetobacter* sp. carries genes that are associated with predicted resistance to carbapenem/oxacillin (Table S2). This may limit the suitability of the isolates for agricultural application due to the potential risk of horizontal transfer of the respective antimicrobial resistance (AMR) genes (Martínez et al. 2015). However, this does not infer the complete dismissal of the isolates as biofertilizer strains. With the array of PGP traits identified in the genomes, a thorough evaluation of the biosafety compliance of the isolates is warranted to prevent discarding promising isolates at the onset of screening. To determine biosafety compliance, further evaluation is necessary to establish whether the AMR is acquired and potentially transferable (located on mobile genetic elements) as intrinsic resistance (chromosomally encoded) is not considered a safety concern. Moreover, phenotypic validation of the predicted AMR profiles through antimicrobial susceptibility testing, such as mean inhibitory concentration assays, are essential to confirm expression of resistance genes and extent of resistance (Rychen et al. 2018). This represents an important direction for future studies on the isolates of interest.

#### *Pseudomonas rhodesiae*

Unlike *C. jiangduensis* and *Acinetobacter* sp., *P. rhodesiae* harbors an array of virulence genes, with a suite of 31 gene hits, as well as a single AMR gene (mexF) (Table S2). The mexF gene forms part of the MexEF-OprN efflux pump system, which is known to contribute to multidrug resistance in clinical *Pseudomonas aeruginosa* strains (Llanes et al. 2011). However, in wild-type strains, the MexEF-OprN operon, unless activated by regulatory mutations, is oftentimes silent (Morita et al. 2015). This suggests that environmental isolates, such as *P. rhodesiae* (isolate55), may also carry the mexF gene without expression, in the absence of similar regulatory mutations. Conversely, if expressed, it is also important to emphasize the ecological role of the MexEF-OprN efflux pump system, which may contribute to the enhancement of *P. rhodesiae’s* competitive advantage, survival and adaptability in the rhizosphere (Alcalde-Rico et al. 2016), which are mandatory traits when prospecting for isolates for biofertilizer development.

Similar to the competitive advantage inferred by the mexF gene, the majority of the virulence genes harbored in *P. rhodesiae* are also frequently associated with persistence, adaptability and effective colonization in the rhizosphere (Table S2). Collectively, genes related to biofilm formation, motility, iron acquisition and adhesion (Table S2) facilitate attachment to roots, stress tolerance and nutrient acquisition, favoring plant-microbe interactions and conferring traits that are necessary for isolates with potential in biofertilizer development (Capdevila et al. 2004; Franklin et al. 2011; Barahona et al. 2016). Additionally, the presence of Type VI secretion system genes (Table S2), may confer a competitive advantage for the isolate. This array of genes and associated traits are increasingly recognized as part of the beneficial gene/trait arsenal in biofertilizer candidates, especially in the genus *Pseudomonas*, which is well known for its multitude of PGP traits including N fixation, P and K mobilization, phytohormone production and antimicrobial compound production (Mehmood et al. 2023; Ghaly et al. 2025). However, certain genes such as the Type VI secretion system genes are sometimes also found in opportunistic strains and may induce virulence (Hood et al. 2010; Coulthurst 2019). This dual role highlights the importance of careful and thorough risk assessment to ensure that beneficial rhizosphere functions are harnessed without inadvertently promoting pathogenicity.

### Literature-supported comparison of traits

Table 3 compares the PGP traits demonstrated by *C. jiangduensis*, *Acinetobacter* sp., and *P. rhodesiae* in published literature, the present study and with the phenotypic insights relayed previously (Akindolire et al. 2025), where the same isolates were evaluated. In essence, the literature search revealed the dearth of information on the application of these isolates. In several instances, mechanistic inferences were required to be made due to the lack of information, emphasizing the urgent need for in planta assays. The genomic insights from this study revealed the array of beneficial traits inherent in the isolates whilst some of these traits were further validated by the phenotypic assay conducted previously (Akindolire et al. 2025). Overall, the table infers that whilst literature does not provide direct evidence for some traits, the combination of the genomic potential and phenotypic validation emphasizes the great potential of these isolates, singly or in combination, as biofertilizer candidates due to their vast potential to improve nutrient acquisition and plant stress tolerance as well as suppress pathogens. However, it is important to also note the presence of AMR and virulence genes in the isolates and conduct the necessary biosafety tests prior to biofertilizer development.

**Table 3:** Comparison of reported biocontrol, nutrient acquisition and abiotic stress tolerance traits in literature versus those observed in *Comamonas jiangduensis*,* Acinetobacter* sp. and *Pseudomonas rhodesiae*

Category	Demonstrated in Literature	Present Study (Genomic insights)	Akindolire et al. (2025) (Phenotypic insights)
*Comamonas jiangduensis* (Isolate31)			
Biocontrol	Direct evidence not available; mechanistic support for biocontrol activity due to biosurfactant production (Sachdev and Cameotra 2013; Sun et al. 2013).	Terpene/ terpene-precursor BGCs (Table 2).	Cellulase activity and biofilm formation.
Nutrient Acquisition	Direct evidence not available; mechanistic support for nutrient acquisition due to biosurfactant production (Sachdev and Cameotra 2013; Sun et al. 2013).	Genes for N acquisition, P solubilization, phytohormone production, germination stimulation, iron acquisition and colonization related proteins (Fig. 2A).	Nitrogen fixation, IAA production and phosphate solubilization.
Abiotic Stress Tolerance	Not reported in literature.	Terpene/ terpene-precursor BGCs (Table 2). Genes for heavy metal and flouride detoxification and neutralizing several abiotic stresses (Fig. 2A and B).	Direct evidence not available on impact on plant growth upon induced abiotic stress however, isolate is cold tolerant (growth observed at 15 °C).
*Acinetobacter* sp. (Isolate33)			
Biocontrol	*Alternaria solani* suppression via hydrogen cyanide, salicylic acid, lytic enzyme and 2,3-dihydroxybenzoic acid production (Danish et al. 2025). *Fusarium oxysporum* suppression via induction of systemic defense through ethylene, salicylic acid, and jasmonic acid signaling pathways (Pisco-Ortiz et al. 2023).	Betalactone, RiPP-like and terpene-precursor BGCs (Table 2) as well as linolenic acid production (Fig. 2A).	Cellulase activity and biofilm formation.
Nutrient Acquisition	Nutrient acquisition via IAA production, phosphate solubilization, and siderophore production (Rokhbakhsh-Zamin et al. 2011; He and Wan 2021).	Genes for P solubilization, metabolism, phytohormone production, germination stimulation, iron acquisition and colonization related proteins (Fig. 2A).	Nitrogen fixation, IAA production and phosphate solubilization.
Abiotic Stress Tolerance	Direct evidence not available; mechanistic support for stress tolerance due to siderophore production (Rokhbakhsh-Zamin et al. 2011).	Arylpolyene and terpene-precursor BGCs (Table 2) as well as linolenic acid production (Fig. 1A). Genes for flouride detoxification, xenobiotics biodegradation and neutralizing several abiotic stresses (Fig. 2A and B).	Direct evidence not available on impact on plant growth upon induced abiotic stress however, isolate is cold tolerant (growth observed at 15 °C).
*Pseudomonas rhodesiae* (Isolate55)			
Biocontrol	Pathogen suppression in rice & cucumber by plant defense activation (Ye et al. 2022; Takeuchi et al. 2024).	NRPS/NRPS-like, betalactone, RiPP-like, NRP-metallophore and terpene-precursor BGCs (Table 2) Genes for efflux pump (MexF) and biofilm formation → rhizosphere competitiveness.	Cellulase and protease activity as well as biofilm formation.
Nutrient Acquisition	Nutrient acquisition via phosphate solubilization, ACC deaminase activity and siderophore and IAA production (Rolón-Cárdenas et al. 2021).	NRPS/NRPS-like, NRP-metallophore and redox-cofactor BGCs (Table 2). Genes for phytohormone production, iron acquisition and P solubilization (Fig. 2A and B).	Nitrogen fixation, phosphate solubilization and IAA and siderophore production.
Abiotic Stress Tolerance	Induced Cd stress tolerance in plant systems (Rolón-Cárdenas et al. 2021, 2022).	NAGGN, redox-cofactor, terpene-precursor and arylpolyene BCGs. Genes for neutralizing several abiotic stresses (Fig. 2A and B).	Direct evidence not available on impact on plant growth upon induced abiotic stress however, isolate is cold tolerant (growth observed at 15 °C).

## Conclusion

This study characterized three psychrotolerant bacterial isolates using comparative genomics, whole-genome annotation and inferred trait analysis. The findings provide genomic evidence for the biocontrol, nutrient acquisition and abiotic stress tolerance capabilities of all isolates, as depicted by PGPg_Finder output and unique BGCs. Overall, all isolates showed promise as candidates for biofertilizer development in sustainable agriculture, particularly in marginal/stress-prone soils or during colder climatic conditions. However, the isolates will need to be interrogated further to conclusively rule out pathogenicity and thereby ensure biosafety. Overall, the integrated analyses provide a robust basis for guiding future experimental assessment and application-oriented studies using the three isolates.

Selected genomic-inferred PGP traits associated with *C. jiangduensis*, *Acinetobacter* sp., and *P. rhodesiae* were validated by the phenotypic insights from our previous work (Akindolire et al. 2025), when evaluating the same isolates. These similarities help in reinforcing the reliability of genome-informed PGP trait predictions. Moreover, this cross-validation between genomic potential and phenotypic traits, supports the use of integrated omics and phenotyping approaches for screening and characterization of PGP microorganisms.

It is important to emphasize that the findings presented here are based on genome-inferred functional potential and prior phenotypic observations, and do not infer direct experimental validation of plant growth-promoting activity under field or in-planta conditions. Future work should therefore focus on evaluating the efficacy of the isolates in planta and under field conditions. Further, the study highlights the vastly underexplored microbial diversity in cold digestate, by the identification of a putative novel *Acinetobacter* sp. with cold tolerance and multiple PGP traits, which adds a new dimension to the bioresource potential of psychrophilic microorganisms. Future work should also therefore be directed towards mining this untapped resource for other potentially promising strains.

## Supplementary Information

Below is the link to the electronic supplementary material.

Supplementary Material 1 (DOCX 1.90 MB)

## Data Availability

The whole genome sequences generated and analysed during the current study are available in the Sequence Read Archive of the National Center for Biotechnology Information (NCBI) repository, with BioSample accession numbers: SAMN54495445-SAMN54495447 under the BioProject accession number PRJNA1258752 ( https:/www.ncbi.nlm.nih.gov/bioproject/PRJNA1258752 ). Additional datasets generated during and/or analysed during the current study are available from the corresponding author on reasonable request.

## References

[CR1] Abd El-Daim IA, Bejai S, Meijer J (2019) Bacillus velezensis 5113 induced metabolic and molecular reprogramming during abiotic stress tolerance in wheat. Sci Rep 9:16282. 10.1038/s41598-019-52567-x 31704956 10.1038/s41598-019-52567-x PMC6841942

[CR2] Akindolire MA, Ndaba B, Bello-Akinosho M et al (2025) Bioprospecting bacteria from psychrophilic anaerobic digestate for potential plant growth-promoting attributes. Int J Microbiol 2025:2208124. 10.1155/ijm/2208124 40313578 10.1155/ijm/2208124 PMC12043392

[CR3] Alcalde-Rico M, Hernando-Amado S, Blanco P, Martínez JL (2016) Multidrug efflux pumps at the crossroad between antibiotic resistance and bacterial virulence. Front Microbiol 7. 10.3389/fmicb.2016.01483 10.3389/fmicb.2016.01483 PMC5030252 27708632

[CR4] Alcock BP, Huynh W, Chalil R et al (2023) CARD 2023: expanded curation, support for machine learning, and resistome prediction at the comprehensive antibiotic resistance database. Nucleic Acids Res 51:D690–D699. 10.1093/nar/gkac920 36263822 10.1093/nar/gkac920 PMC9825576

[CR5] Ali A, Dindhoria K, Kumar R (2023) *Acinetobacter oleivorans* IRS14 alleviates cold stress in wheat by regulating physiological and biochemical factors. J Appl Microbiol 134:lxad176. 10.1093/jambio/lxad176 37550224 10.1093/jambio/lxad176

[CR6] Andrews S (2010) FastQC: a quality control tool for high throughput sequence data

[CR7] Arkin AP, Cottingham RW, Henry CS et al (2018) KBase: the United States department of energy systems biology knowledgebase. Nat Biotechnol 36:566–569. 10.1038/nbt.4163 29979655 10.1038/nbt.4163 PMC6870991

[CR8] Arnison PG, Bibb MJ, Bierbaum G et al (2013) Ribosomally synthesized and post-translationally modified peptide natural products: overview and recommendations for a universal nomenclature. Nat Prod Rep 30:108–160. 10.1039/C2NP20085F 23165928 10.1039/c2np20085f PMC3954855

[CR9] Bankevich A, Nurk S, Antipov D et al (2012) SPAdes: a new genome assembly algorithm and its applications to single-cell sequencing. J Comput Biol 19:455–477. 10.1089/cmb.2012.0021 22506599 10.1089/cmb.2012.0021 PMC3342519

[CR10] Barahona E, Navazo A, Garrido-Sanz D et al (2016) Pseudomonas fluorescens F113 can produce a second flagellar apparatus, which is important for plant root colonization. Front Microbiol 7. 10.3389/fmicb.2016.01471 10.3389/fmicb.2016.01471 PMC5031763 27713729

[CR11] Bashiri G, Antoney J, Jirgis ENM et al (2019) A revised biosynthetic pathway for the cofactor F420 in prokaryotes. Nat Commun 10:1558. 10.1038/s41467-019-09534-x 30952857 10.1038/s41467-019-09534-x PMC6450877

[CR13] Blin K, Shaw S, Kloosterman AM et al (2021) antiSMASH 6.0: improving cluster detection and comparison capabilities. Nucleic Acids Res 49:W29–W35. 10.1093/nar/gkab335 33978755 10.1093/nar/gkab335 PMC8262755

[CR12] Blin K, Shaw S, Augustijn HE et al (2023) antiSMASH 7.0: new and improved predictions for detection, regulation, chemical structures and visualisation. Nucleic Acids Res 51:W46–W50. 10.1093/nar/gkad344 37140036 10.1093/nar/gkad344 PMC10320115

[CR14] Bolger AM, Lohse M, Usadel B (2014) Trimmomatic: a flexible trimmer for Illumina sequence data. Bioinformatics 30:2114–2120. 10.1093/bioinformatics/btu170 24695404 10.1093/bioinformatics/btu170 PMC4103590

[CR15] Buchfink B, Xie C, Huson DH (2015) Fast and sensitive protein alignment using DIAMOND. Nat Methods 12:59–60. 10.1038/nmeth.3176 25402007 10.1038/nmeth.3176

[CR16] Capdevila S, Martínez-Granero FM, Sánchez-Contreras M et al (2004) Analysis of Pseudomonas fluorescens F113 genes implicated in flagellar filament synthesis and their role in competitive root colonization. Microbiology 150:3889–3897. 10.1099/mic.0.27362-0 15528673 10.1099/mic.0.27362-0

[CR17] Chaumeil P-A, Mussig AJ, Hugenholtz P, Parks DH (2020) GTDB-Tk: a toolkit to classify genomes with the Genome Taxonomy Database. Bioinformatics 36:1925–1927. 10.1093/bioinformatics/btz848 10.1093/bioinformatics/btz848 PMC7703759 31730192

[CR18] Chen H, Boutros PC (2011) VennDiagram: a package for the generation of highly-customizable Venn and Euler diagrams in R. BMC Bioinformatics 12:35. 10.1186/1471-2105-12-35 21269502 10.1186/1471-2105-12-35 PMC3041657

[CR20] Chen L, Zheng D, Liu B et al (2016) Hierarchical and refined dataset for big data analysis—10 years on. Nucleic Acids Res 44:D694–D697. 10.1093/nar/gkv1239 26578559 10.1093/nar/gkv1239 PMC4702877

[CR21] Chen R, Wong HL, Burns BP (2019) New approaches to detect biosynthetic gene clusters in the environment. Medicines 6:32. 10.3390/medicines6010032 30823559 10.3390/medicines6010032 PMC6473659

[CR19] Chen J, Feng Y, Ma J et al (2025) Genomic and metabolomic insights into the antimicrobial compounds and plant growth-promoting potential of Bacillus velezensis B115. Sci Rep 15:10666. 10.1038/s41598-025-92322-z 40148367 10.1038/s41598-025-92322-z PMC11950384

[CR22] Choi O, Kim J, Kim J-G et al (2008) Pyrroloquinoline Quinone is a plant growth promotion factor produced by *Pseudomonas fluorescens* B16. Plant Physiol 146:657–668. 10.1104/pp.107.112748 18055583 10.1104/pp.107.112748 PMC2245851

[CR23] Chun J, Oren A, Ventosa A et al (2018) Proposed minimal standards for the use of genome data for the taxonomy of prokaryotes. Int J Syst Evol MicroBiol 68:461–466. 10.1099/ijsem.0.002516 29292687 10.1099/ijsem.0.002516

[CR24] Cimermancic P, Medema MH, Claesen J et al (2014) Insights into Secondary Metabolism from a Global Analysis of Prokaryotic Biosynthetic Gene Clusters. Cell 158:412–421. 10.1016/j.cell.2014.06.034 25036635 10.1016/j.cell.2014.06.034 PMC4123684

[CR25] Coulthurst S (2019) The Type VI secretion system: a versatile bacterial weapon. Microbiology 165:503–515. 10.1099/mic.0.000789 30893029 10.1099/mic.0.000789

[CR26] Crits-Christoph A, Diamond S, Butterfield CN et al (2018) Novel soil bacteria possess diverse genes for secondary metabolite biosynthesis. Nature 558:440–444. 10.1038/s41586-018-0207-y 29899444 10.1038/s41586-018-0207-y

[CR27] Danish M, Shahid M, Shafi Z et al (2025) Boosting disease resistance in Solanum melongena L. (eggplant) against Alternaria solani: the synergistic effect of biocontrol Acinetobacter sp. and indole-3-acetic acid (IAA). World J Microbiol Biotechnol 41:85. 10.1007/s11274-025-04282-1 40011313 10.1007/s11274-025-04282-1

[CR28] Douka D, T-N Spantidos, P Katinakis, A Venieraki (2025) Unveiling the genomic basis of antagonism and plant growth promotion in the novel endophyte bacillus velezensis strain B.B.Sf.2. DNA 5:23. 10.3390/dna5020023

[CR29] Duong TT, Nguyen TTL, Dinh THV et al (2021) Auxin production of the filamentous cyanobacterial Planktothricoides strain isolated from a polluted river in Vietnam. Chemosphere 284:131242. 10.1016/j.chemosphere.2021.131242 34225111 10.1016/j.chemosphere.2021.131242

[CR30] Eng F, Marin JE, Zienkiewicz K et al (2021) Jasmonic acid biosynthesis by fungi: derivatives, first evidence on biochemical pathways and culture conditions for production. PeerJ 9:e10873. 10.7717/peerj.10873 33604199 10.7717/peerj.10873 PMC7869668

[CR31] Eren AM, Esen ÖC, Quince C et al (2015) Anvi’o: an advanced analysis and visualization platform for ‘omics data. PeerJ 3:e1319. 10.7717/peerj.1319 26500826 10.7717/peerj.1319 PMC4614810

[CR32] Fadiji AE, Xiong C, Egidi E, Singh BK (2024) Formulation challenges associated with microbial biofertilizers in sustainable agriculture and paths forward. J Sust Agri Env 3:e70006. 10.1002/sae2.70006

[CR33] Feldgarden M, Brover V, Gonzalez-Escalona N et al (2021) AMRFinderPlus and the Reference Gene Catalog facilitate examination of the genomic links among antimicrobial resistance, stress response, and virulence. Sci Rep 11:12728. 10.1038/s41598-021-91456-0 34135355 10.1038/s41598-021-91456-0 PMC8208984

[CR34] Franklin MJ, Nivens DE, Weadge JT, Howell PL (2011) Biosynthesis of the pseudomonas aeruginosa extracellular polysaccharides, alginate, Pel, and Psl. Front Microbio 2. 10.3389/fmicb.2011.00167 10.3389/fmicb.2011.00167 PMC3159412 21991261

[CR35] Ghaly TM, Fabian BK, Vick SHW et al (2025) Genetic Drivers of Plant Root Colonisation by the Biocontrol Agent *Pseudomonas protegens* Pf-5. Environ Microbiol Rep 17:e70179. 10.1111/1758-2229.70179 40827064 10.1111/1758-2229.70179 PMC12361813

[CR36] Grant JR, Enns E, Marinier E et al (2023) Proksee: in-depth characterization and visualization of bacterial genomes. Nucleic Acids Res 51:W484–W492. 10.1093/nar/gkad326 37140037 10.1093/nar/gkad326 PMC10320063

[CR37] He D, Wan W (2021) Phosphate-Solubilizing Bacterium Acinetobacter pittii gp-1 Affects Rhizosphere Bacterial Community to Alleviate Soil Phosphorus Limitation for Growth of Soybean (Glycine max). Front Microbiol 12:737116. 10.3389/fmicb.2021.737116 34630363 10.3389/fmicb.2021.737116 PMC8498572

[CR38] Hibbing ME, Fuqua C, Parsek MR, Peterson SB (2010) Bacterial competition: surviving and thriving in the microbial jungle. Nat Rev Microbiol 8:15–25. 10.1038/nrmicro2259 19946288 10.1038/nrmicro2259 PMC2879262

[CR39] Hood RD, Singh P, Hsu F et al (2010) A Type VI Secretion System of Pseudomonas aeruginosa Targets a Toxin to Bacteria. Cell Host Microbe 7:25–37. 10.1016/j.chom.2009.12.007 20114026 10.1016/j.chom.2009.12.007 PMC2831478

[CR40] Hyatt D, Chen G-L, LoCascio PF et al (2010) Prodigal: prokaryotic gene recognition and translation initiation site identification. BMC Bioinformatics 11:119. 10.1186/1471-2105-11-119 20211023 10.1186/1471-2105-11-119 PMC2848648

[CR41] Jain C, Rodriguez-R LM, Phillippy AM et al (2018) High throughput ANI analysis of 90K prokaryotic genomes reveals clear species boundaries. Nat Commun 9:5114. 10.1038/s41467-018-07641-9 30504855 10.1038/s41467-018-07641-9 PMC6269478

[CR42] Kang SH, Cho H-S, Cheong H et al (2007) Two bacterial entophytes eliciting both plant growth promotion and plant defense on pepper (Capsicum annuum L). J Microbiol Biotechnol 17:96–103 18051359

[CR43] Kolde R (2015) Package ‘pheatmap.’ R package 1:790

[CR44] Kumar P, Rani S, Dahiya P et al (2022) Whole genome analysis for plant growth promotion profiling of Pantoea agglomerans CPHN2, a non-rhizobial nodule endophyte. Front Microbiol 13:998821. 10.3389/fmicb.2022.998821 36419432 10.3389/fmicb.2022.998821 PMC9676466

[CR45] Lee MD (2019) GToTree: a user-friendly workflow for phylogenomics. Bioinformatics 35:4162–4164. 10.1093/bioinformatics/btz188 30865266 10.1093/bioinformatics/btz188 PMC6792077

[CR46] Letunic I, Bork P (2021) Interactive Tree Of Life (iTOL) v5: an online tool for phylogenetic tree display and annotation. Nucleic Acids Res 49:W293–W296. 10.1093/nar/gkab301 33885785 10.1093/nar/gkab301 PMC8265157

[CR47] Llanes C, Köhler T, Patry I et al (2011) Role of the MexEF-OprN Efflux System in Low-Level Resistance of Pseudomonas aeruginosa to Ciprofloxacin. Antimicrob Agents Chemother 55:5676–5684. 10.1128/AAC.00101-11 21911574 10.1128/AAC.00101-11 PMC3232816

[CR48] Martínez JL, Coque TM, Baquero F (2015) What is a resistance gene? Ranking risk in resistomes. Nat Rev Microbiol 13:116–123. 10.1038/nrmicro3399 25534811 10.1038/nrmicro3399

[CR49] Medema MH, Blin K, Cimermancic P et al (2011) antiSMASH: rapid identification, annotation and analysis of secondary metabolite biosynthesis gene clusters in bacterial and fungal genome sequences. Nucleic Acids Res 39:W339–W346. 10.1093/nar/gkr466 21672958 10.1093/nar/gkr466 PMC3125804

[CR50] Medema MH, Kottmann R, Yilmaz P et al (2015) Minimum Information about a Biosynthetic Gene cluster. Nat Chem Biol 11:625–631. 10.1038/nchembio.1890 26284661 10.1038/nchembio.1890 PMC5714517

[CR51] Mehmood N, Saeed M, Zafarullah S et al (2023) Multifaceted Impacts of Plant-Beneficial *Pseudomonas* spp. in Managing Various Plant Diseases and Crop Yield Improvement. ACS Omega 8:22296–22315. 10.1021/acsomega.3c00870 37396244 10.1021/acsomega.3c00870 PMC10308577

[CR52] Meier-Kolthoff JP, Göker M (2019) TYGS is an automated high-throughput platform for state-of-the-art genome-based taxonomy. Nat Commun 10:2182. 10.1038/s41467-019-10210-3 31097708 10.1038/s41467-019-10210-3 PMC6522516

[CR53] Miethke M, Marahiel MA (2007) Siderophore-Based Iron Acquisition and Pathogen Control. Microbiol Mol Biol Rev 71:413–451. 10.1128/MMBR.00012-07 17804665 10.1128/MMBR.00012-07 PMC2168645

[CR54] Mitter EK, Tosi M, Obregón D et al (2021) Rethinking Crop Nutrition in Times of Modern Microbiology: Innovative Biofertilizer Technologies. Front Sustain Food Syst 5:606815. 10.3389/fsufs.2021.606815

[CR55] Morita Y, Tomida J, Kawamura Y (2015) Efflux-mediated fluoroquinolone resistance in the multidrug-resistant Pseudomonas aeruginosa clinical isolate PA7: identification of a novel MexS variant involved in upregulation of the mexEF-oprN multidrug efflux operon. Front Microbiol 6. 10.3389/fmicb.2015.00008 10.3389/fmicb.2015.00008 PMC4301020 25653649

[CR56] Mujumdar S, Bhoyar J, Akkar A et al (2023) Acinetobacter: A versatile plant growth-promoting rhizobacteria (PGPR). In: Swapnil P, Meena M, Harish A (eds), Plant-Microbe Interaction - Recent Advances in Molecular and Biochemical Approaches. Elsevier, pp 327–362 10.1016/B978-0-323-91875-6.00009-8

[CR57] Nabati J, Nezami A, Yousefi A et al (2025) Biofertilizers containing plant growth promoting rhizobacteria enhance nutrient uptake and improve the growth and yield of chickpea plants in an arid environment. Sci Rep 15:8331. 10.1038/s41598-025-93070-w 40065116 10.1038/s41598-025-93070-w PMC11894201

[CR58] Ongena M, Jacques P (2008) Bacillus lipopeptides: versatile weapons for plant disease biocontrol. Trends Microbiol 16:115–125. 10.1016/j.tim.2007.12.009 18289856 10.1016/j.tim.2007.12.009

[CR59] Pandey P, Irulappan V, Bagavathiannan MV, Senthil-Kumar M (2017) Impact of combined abiotic and biotic stresses on plant growth and avenues for crop improvement by exploiting physio-morphological traits. Front Plant Sci 8. 10.3389/fpls.2017.00537 10.3389/fpls.2017.00537 PMC5394115 28458674

[CR60] Parks DH, Imelfort M, Skennerton CT et al (2015) CheckM: assessing the quality of microbial genomes recovered from isolates, single cells, and metagenomes. Genome Res 25:1043–1055. 10.1101/gr.186072.114 25977477 10.1101/gr.186072.114 PMC4484387

[CR61] Patakova P, Vasylkivska M, Sedlar K et al (2024) Whole genome sequencing and characterization of Pantoea agglomerans DBM 3797, endophyte, isolated from fresh hop (Humulus lupulus L). Front Microbiol 15:1305338. 10.3389/fmicb.2024.1305338 38389535 10.3389/fmicb.2024.1305338 PMC10882544

[CR62] Patz S, Gautam A, Becker M et al (2021) PLaBAse: A comprehensive web resource for analyzing the plant growth-promoting potential of plant-associated bacteria

[CR63] Pellegrinetti TA, Monteiro GGTN, Lemos LN et al (2024) PGPg_finder: A comprehensive and user-friendly pipeline for identifying plant growth-promoting genes in genomic and metagenomic data. Rhizosphere 30:100905. 10.1016/j.rhisph.2024.100905

[CR64] Pisco-Ortiz C, González-Almario A, Uribe-Gutiérrez L et al (2023) Suppression of tomato wilt by cell-free supernatants of Acinetobacter baumannii isolates from wild cacao from the Colombian Amazon. World J Microbiol Biotechnol 39:297. 10.1007/s11274-023-03719-9 37658991 10.1007/s11274-023-03719-9 PMC10475004

[CR65] Prjibelski A, Antipov D, Meleshko D et al (2020) Using SPAdes De Novo Assembler. CP Bioinf 70:e102. 10.1002/cpbi.102 10.1002/cpbi.102 32559359

[CR66] Raimi A, Adeleke R (2023) 16S rRNA gene-based identification and plant growth‐promoting potential of cultivable endophytic bacteria. Agron J 115:1447–1462. 10.1002/agj2.21241

[CR67] Riesco R, Trujillo ME (2024) Update on the proposed minimal standards for the use of genome data for the taxonomy of prokaryotes. Int J Syst Evol MicroBiol 74. 10.1099/ijsem.0.006300 10.1099/ijsem.0.006300 PMC10963913 38512750

[CR68] Rikame T, Borde M (2022) Whole genome, functional annotation and comparative genomics of plant growth-promoting bacteria pseudomonas aeruginosa (NG61) with potential application in agro-industry. Curr Microbiol 79:169. 10.1007/s00284-022-02845-1 35460384 10.1007/s00284-022-02845-1

[CR69] Robinson SL, Christenson JK, Wackett LP (2019) Biosynthesis and chemical diversity of β-lactone natural products. Nat Prod Rep 36:458–475. 10.1039/C8NP00052B 30191940 10.1039/c8np00052b

[CR70] Rodríguez-Gijón A, Buck M, Andersson AF et al (2023) Linking prokaryotic genome size variation to metabolic potential and environment. ISME Commun 3:25. 10.1038/s43705-023-00231-x 36973336 10.1038/s43705-023-00231-x PMC10042847

[CR71] Rokhbakhsh-Zamin F, Sachdev D, Kazemi-Pour N et al (2011) Characterization of plant-growth-promoting traits of Acinetobacter species isolated from rhizosphere of Pennisetum glaucum. J Microbiol Biotechnol 21:556–566 21715961

[CR72] Rolón-Cárdenas GA, Arvizu-Gómez JL, Pacheco-Aguilar JR et al (2021) Cadmium-tolerant endophytic Pseudomonas rhodesiae strains isolated from Typha latifolia modify the root architecture of Arabidopsis thaliana Col-0 in presence and absence of Cd. Braz J Microbiol 52:349–361. 10.1007/s42770-020-00408-9 33236245 10.1007/s42770-020-00408-9 PMC7966613

[CR73] Rolón-Cárdenas GA, Martínez-Martínez JG, Arvizu-Gómez JL et al (2022) Enhanced Cd-Accumulation in Typha latifolia by Interaction with Pseudomonas rhodesiae GRC140 under Axenic Hydroponic Conditions. Plants 11:1447. 10.3390/plants11111447 35684220 10.3390/plants11111447 PMC9183143

[CR74] Rychen G, Azimonti G, Bampidis V et al (2018) Guidance on the characterisation of microorganisms used as feed additives or as production organisms. EFS2 16. 10.2903/j.efsa.2018.5206 10.2903/j.efsa.2018.5206 PMC7009341 32625840

[CR75] Sachdev DP, Cameotra SS (2013) Biosurfactants in agriculture. Appl Microbiol Biotechnol 97:1005–1016. 10.1007/s00253-012-4641-8 23280539 10.1007/s00253-012-4641-8 PMC3555348

[CR76] Sagot B, Gaysinski M, Mehiri M et al (2010) Osmotically induced synthesis of the dipeptide N-acetylglutaminylglutamine amide is mediated by a new pathway conserved among bacteria. Proc Natl Acad Sci USA 107:12652–12657. 10.1073/pnas.1003063107 20571117 10.1073/pnas.1003063107 PMC2906558

[CR77] Sahoo A, Yadav G, Mehta T et al (2025) Omics-driven insights into plant growth-promoting microorganisms for sustainable agriculture. Discov Sustain 6:659. 10.1007/s43621-025-01582-2

[CR78] Seemann T (2014) Prokka: rapid prokaryotic genome annotation. Bioinformatics 30:2068–2069. 10.1093/bioinformatics/btu153 24642063 10.1093/bioinformatics/btu153

[CR79] Seemann T (2016) ABRicate: mass screening of contigs for antiobiotic resistance genes

[CR80] Shaffer M, Borton MA, McGivern BB et al (2020) DRAM for distilling microbial metabolism to automate the curation of microbiome function. Nucleic Acids Res 48:8883–8900. 10.1093/nar/gkaa621 32766782 10.1093/nar/gkaa621 PMC7498326

[CR81] Sharma P, Pandey R, Chauhan NS (2024) Biofertilizer and biocontrol properties of Stenotrophomonas maltophilia BCM emphasize its potential application for sustainable agriculture. Front Plant Sci 15:1364807. 10.3389/fpls.2024.1364807 38501138 10.3389/fpls.2024.1364807 PMC10944936

[CR82] Singh V, Kumar B (2024) A review of agricultural microbial inoculants and their carriers in bioformulation. Rhizosphere 29:100843. 10.1016/j.rhisph.2023.100843

[CR84] Sun L-N, Zhang J, Chen Q et al (2013) Comamonas jiangduensis sp. nov., a biosurfactant-producing bacterium isolated from agricultural soil. Int J Syst Evol MicroBiol 63:2168–2173. 10.1099/ijs.0.045716-0 23125317 10.1099/ijs.0.045716-0

[CR83] Sun H, Levenfors JJ, Brandt C, Schnürer A (2025) Assessing phenotypic and genotypic antibiotic resistance in bacillus-related bacteria isolated from biogas digestates. Ecotoxicol Environ Saf 291:117859. 10.1016/j.ecoenv.2025.117859 39947064 10.1016/j.ecoenv.2025.117859

[CR85] Takeuchi K, Ogiso M, Ota A et al (2024) Pseudomonas rhodesiae HAI-0804 suppresses Pythium damping off and root rot in cucumber by its efficient root colonization promoted by amendment with glutamate. Front Microbiol 15:1485167. 10.3389/fmicb.2024.1485167 39564481 10.3389/fmicb.2024.1485167 PMC11573540

[CR86] Vassileva M, Mocali S, Canfora L et al (2022) Safety Level of Microorganism-Bearing Products Applied in Soil-Plant Systems. Front Plant Sci 13:862875. 10.3389/fpls.2022.862875 35574066 10.3389/fpls.2022.862875 PMC9096872

[CR87] Wang C, Zhao X, Wu K et al (2023) Isolation and characterization of Bacillus velezensis strain B19 for biocontrol of Panax notoginseng root rot. Biol Control 185:105311. 10.1016/j.biocontrol.2023.105311

[CR88] Wang Y, Dall’Agnol RF, Bertani I et al (2024) Identification of synthetic consortia from a set of plant-beneficial bacteria. Microb Biotechnol 17:e14330. 10.1111/1751-7915.14330 38291799 10.1111/1751-7915.14330 PMC10884989

[CR89] Wickham H (2011) ggplot2. WIREs Comput Stats 3:180–185. 10.1002/wics.147

[CR90] Yamada Y, Kuzuyama T, Komatsu M et al (2015) Terpene synthases are widely distributed in bacteria. Proc Natl Acad Sci USA 112:857–862. 10.1073/pnas.1422108112 25535391 10.1073/pnas.1422108112 PMC4311827

[CR91] Ye S, Yan R, Li X et al (2022) Biocontrol potential of Pseudomonas rhodesiae GC-7 against the root-knot nematode Meloidogyne graminicola through both antagonistic effects and induced plant resistance. Front Microbiol 13:1025727. 10.3389/fmicb.2022.1025727 36386722 10.3389/fmicb.2022.1025727 PMC9651087

[CR92] Zhang S, Li X, Wu J et al (2021) Molecular Methods for Pathogenic Bacteria Detection and Recent Advances in Wastewater Analysis. Water 13:3551. 10.3390/w13243551

[CR93] Zhang Z, Zhang L, Zhang L et al (2024) Diversity and distribution of biosynthetic gene clusters in agricultural soil microbiomes. mSystems 9:e01263–e01223. 10.1128/msystems.01263-23 38470142 10.1128/msystems.01263-23 PMC11019929

[CR94] Zhao G, Zhu X, Zheng G et al (2024) Development of biofertilizers for sustainable agriculture over four decades (1980–2022). Geogr Sustain 5:19–28. 10.1016/j.geosus.2023.09.006

