# Supplementary material for: Plant growth promoting traits of selected psychrotolerant bacteria: a genomic basis of biocontrol, nutrient acquisition and stress tolerance
Source: World J Microbiol Biotechnol. 2026 May 8;42(6):266. doi: 10.1007/s11274-026-04994-y (PMC13156110; doi:10.1007/s11274-026-04994-y)
Supplement: Supplementary file 1 — Supplementary Material 1 (DOCX 1.90 MB) [file 11274_2026_4994_MOESM1_ESM.docx]

**Plant Growth Promoting Traits of Selected Psychrotolerant Bacteria: A Genomic Basis of** **Biocontrol, Nutrient Acquisition and Stress Tolerance**

Ashira Roopnarain^1,2*^, Muyiwa Ajoke Akindolire^1^, Thierry Alexandre Pellegrinetti^3^, Haripriya Rama^1*^

^1^ Microbiology and Environmental Biotechnology Research Group, Agricultural Research Council - Natural Resources and Engineering, Pretoria, South Africa

^2^ Department of Environmental Sciences, College of Agriculture and Environmental Sciences, University of South Africa – Florida Campus, Private Bag X6, Florida, 1710, South Africa

^3^ Département de phytologie, Faculté des sciences de l'agriculture et de l'alimentation, Université Laval, Québec City Québec, Canada

**Supplementary information**


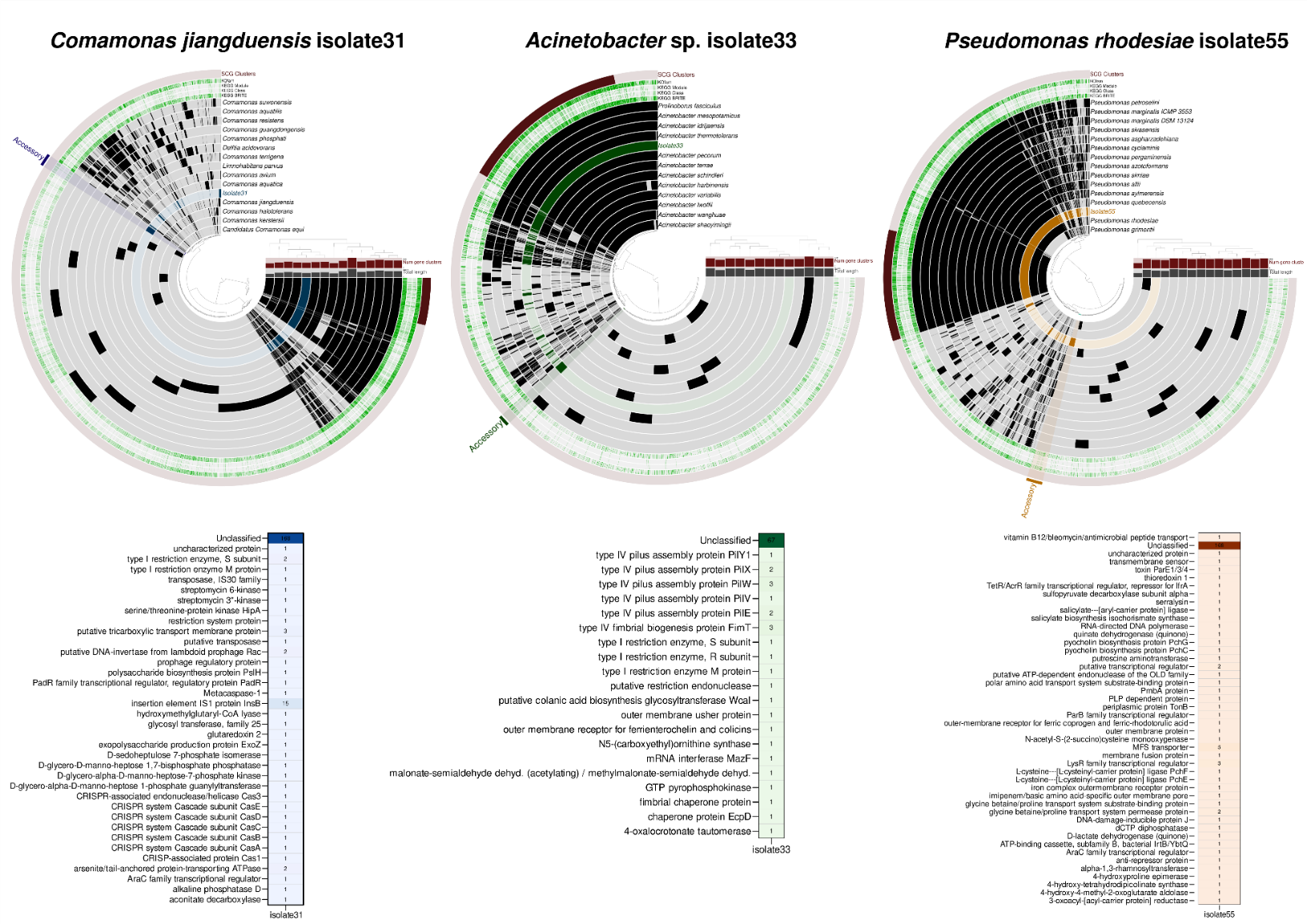


**Figure S1**: Comparative pangenome analysis of the studied isolates and closely related reference genomes. Gene clusters are partitioned into core genes shared by all analysed genomes, accessory genes shared by a subset of genomes, and unique genes specific to individual isolates. This pan-genome structure reflects both shared and isolate-specific genetic potential, highlighting genomic diversity and differentiation among the strains.


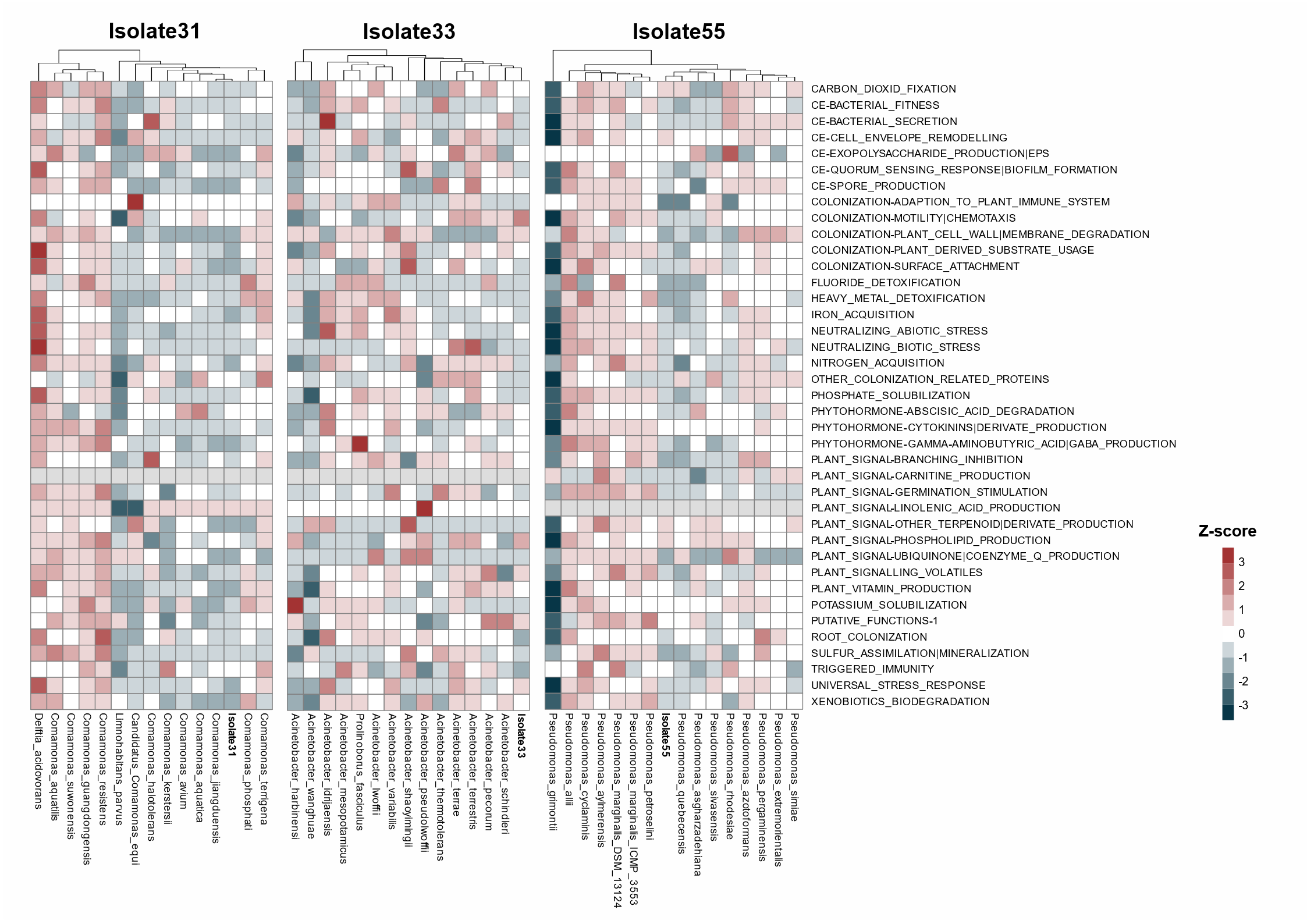


**Figure S2**: Comparative PGPg_Finder visualization of plant growth-promoting functional categories in the studied isolates and closely related reference strains. Colour intensity reflects relative gene counts per functional category and indicates genomic representation rather than functional activity or performance.


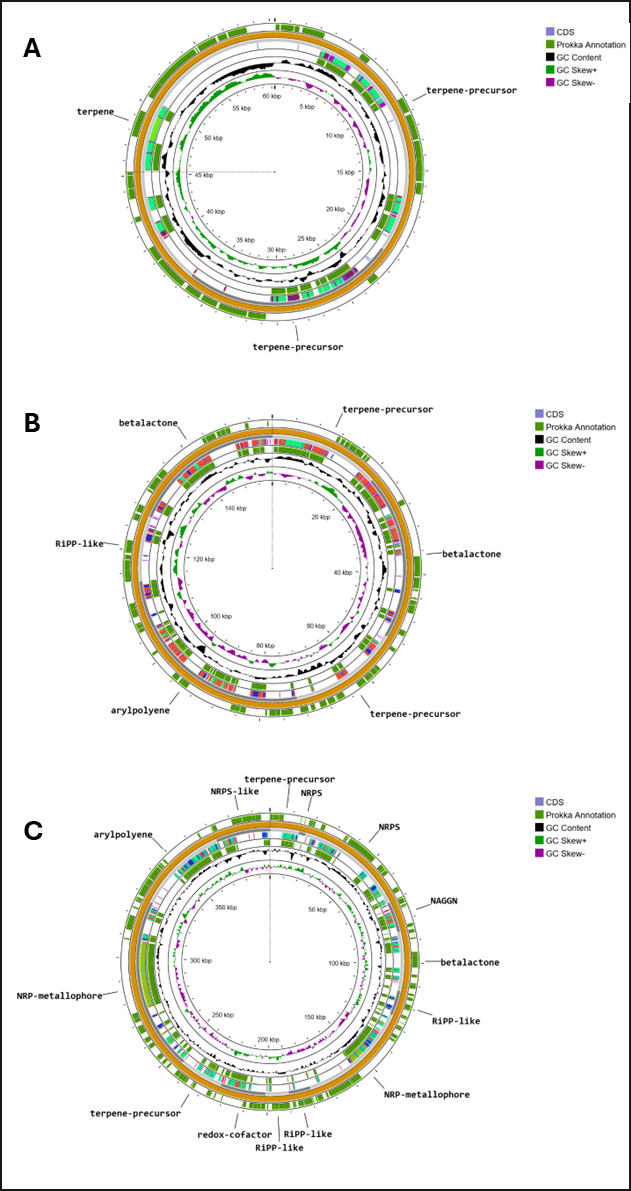


**Figure S3:** Circular genome maps of (A) isolate31 (*Comamonas jiangduensis*) (B) isolate33 (*Acinetobacter* sp.) and (C) isolate55 (*Pseudomonas rhodesiae*) showing biosynthetic gene clusters predicted by antiSMASH and visualized using Proksee.

**Table S1**: Genome quality, taxonomy, and functional annotation

| **Isolate** | **Genome size (bp)** | **Contigs** | **Completeness (%)** | **Contamination (%)** | **GTDB-Tk classification** | **Closest GTDB reference** | **Taxonomic confidence** | **DRAM functional highlights*** |
| --- | --- | --- | --- | --- | --- | --- | --- | --- |
| Isolate 31 | 3,388,837 | 30 | 98.28 | 0.83 | d__Bacteria;  p__Proteobacteria;  c__Gammaproteobacteria;  o__Burkholderiales;  f__Burkholderiaceae;  g__*Comamonas*;  s__ *Comamonas jiangduensis* | GCF_902829245.1 | Species-level | Carbohydrate utilization including galactose and mannose degradation (Woodcroft); ABC-type nutrient transporters, including phosphonate and iron-related transport signatures; energy/redox metabolism. |
| Isolate 33 | 3,053,062 | 29 | 99.66 | 0.07 | d__Bacteria;  p__Proteobacteria;  c__Gammaproteobacteria;  o__Pseudomonadales;  f__Moraxellaceae;  g__*Acinetobacter*;  s__ | *GCA_013530545.1, s__Acinetobacter lwof...* | Below species threshold | Carbohydrate utilization with galactose degradation (Woodcroft); ABC-type nutrient transporters; betaine biosynthesis from choline; energy/redox metabolism. |
| Isolate 55 | 5,765,167 | 21 | 100.0 | 0.11 | d__Bacteria;  p__Proteobacteria;  c__Gammaproteobacteria;  o__Pseudomonadales;  f__Pseudomonadaceae;  g__*Pseudomonas*_E;  s__*Pseudomonas*_E *rhodesiae* | GCF_007858255.1 | Species-level | Expanded carbohydrate repertoire including galactose, galacturonic acid, xylose and fructose degradation (Woodcroft); enriched CAZy functions including GH13 starch backbone cleavage and alginate lyase-associated PL5/PL7; diverse ABC-type transport systems and iron-related transport signatures; betaine biosynthesis from choline; energy/redox metabolism. |

*Functional highlights are summarised from DRAM metabolism_summary outputs and represent predicted metabolic potential rather than demonstrated activity.

**Table S2**: Comprehensive ABRicate analysis of resistance and virulence-associated genes in *Comamonas jiangduensis*, *Acinetobacter* sp. and *Pseudomonas rhodesiae* using CARD, NCBI, and VFDB databases.

| **Database** | **Hits/ Count** | **Gene Hits (Identity %)** | **Potential Role** | **Reference(s)** |
| --- | --- | --- | --- | --- |
| *Comamonas jiangduensis* | | | | |
| CARD | 3 | ANT(3'')-IIa (99.28%),  APH(3'')-Ib (100%), APH(6)-Id (100%) | Aminoglycoside-modifying enzymes (ANT(3'')-IIa, APH(3'')-Ib, APH(6)-Id) → aminoglycoside resistance; AMR dissemination risk | Van Hoek et al. (2011) |
| NCBI | 3 | aadA1 (100%), aph(3'')-Ib (100%), aph(6)-Id (100%) | Aminoglycoside resistance; AMR dissemination risk | Van Hoek et al. (2011) |
| VFDB | 0 | None detected | - | - |
| *Acinetobacter* sp. | | | | |
| CARD | 1 | OXA-285 (100%) | β-lactamase (OXA-285) → hydrolyzes carbapenems/oxacillin; carbapenem/oxacillin resistance | Evans and Amyes (2014) |
| NCBI | 1 | blaOXA-646 (97.74%) | β-lactamase (blaOXA-646) → clinically relevant AMR determinant | Evans and Amyes (2014) |
| VFDB | 0 | None detected | - | - |
| *Pseudomonas rhodesiae* | | | | |
| CARD | 1 | MexF (98.75%) | Efflux pump (MexF) → multidrug resistance | Li et al. (1998) |
| NCBI | 0 | None detected | - | - |
| VFDB | 31 | alg8 (97.85%),  algA (97.1%),  algI (82.21%),  algU (98.11%), clpV1 (98.12%), dotU1 (93.56%), fleN (95.85%),  fleQ (99.93%),  flgC (99.77%),  flgG (98.09%),  flgH (89.8%),  flgI (94.77%),  flhA (99.39%),  fliA (91.53%),  fliG (99.71%),  fliI (95.06%),  fliM (96.19%),  fliN (91.98%),  fliP (92.19%),  fliQ (99.63%),  hcp1 (95.91%), hsiB1/vipA (91.33%), hsiC1/vipB (97.46%),  hsiG1 (99.68%), mbtH-like (92.69%), pilG (96.81%),  pilH (100%),  pvdH (94.4%),  pvdO (90.29%), pvdS (90.78%), waaF (93.55%) | Biofilm production; stress adaptation/tolerance (alg8, algA, algI, algU);  Motility root colonization (fleN, fleQ, flgC, flgG, flgH, flgI, flhA, fliA, fliG, fliI, fliM, fliN, fliP, fliQ);  Type VI secretion system; microbial competition, virulence and niche adaptation (clpV1, dotU1, hcp1, hsiB1/vipA, hsiC1/vipB, hsiG1);  Iron acquisition; iron scavenging and siderophore production (mbtH-like, pvdH, pvdO, pvdS);  Adhesion; surface attachment and biofilm formation (pilG, pilH);  Cell envelope integrity; outer membrane stability (waaF) | De Kievit and Lam (1997); Capdevila et al. (2004); Hood et al. (2010); Franklin et al. (2011); Persat et al. (2015); Barahona et al. (2016); Coulthurst (2019); Bonneau et al. (2020) |

**References**

Barahona E, Navazo A, Garrido-Sanz D, et al (2016) Pseudomonas fluorescens F113 Can Produce a Second Flagellar Apparatus, Which Is Important for Plant Root Colonization. Front Microbiol 7:. https://doi.org/10.3389/fmicb.2016.01471

Bonneau A, Roche B, Schalk IJ (2020) Iron acquisition in Pseudomonas aeruginosa by the siderophore pyoverdine: an intricate interacting network including periplasmic and membrane proteins. Sci Rep 10:120. https://doi.org/10.1038/s41598-019-56913-x

Capdevila S, Martínez-Granero FM, Sánchez-Contreras M, et al (2004) Analysis of Pseudomonas fluorescens F113 genes implicated in flagellar filament synthesis and their role in competitive root colonization. Microbiology 150:3889–3897. https://doi.org/10.1099/mic.0.27362-0

Coulthurst S (2019) The Type VI secretion system: a versatile bacterial weapon. Microbiology 165:503–515. https://doi.org/10.1099/mic.0.000789

De Kievit TR, Lam JS (1997) Isolation and characterization of two genes, waaC (rfaC) and waaF (rfaF), involved in Pseudomonas aeruginosa serotype O5 inner-core biosynthesis. J Bacteriol 179:3451–3457. https://doi.org/10.1128/jb.179.11.3451-3457.1997

Evans BA, Amyes SGB (2014) OXA β-Lactamases. Clin Microbiol Rev 27:241–263. https://doi.org/10.1128/CMR.00117-13

Franklin MJ, Nivens DE, Weadge JT, Howell PL (2011) Biosynthesis of the Pseudomonas aeruginosa Extracellular Polysaccharides, Alginate, Pel, and Psl. Front Microbio 2:. https://doi.org/10.3389/fmicb.2011.00167

Hood RD, Singh P, Hsu F, et al (2010) A Type VI Secretion System of Pseudomonas aeruginosa Targets a Toxin to Bacteria. Cell Host & Microbe 7:25–37. https://doi.org/10.1016/j.chom.2009.12.007

Li X-Z, Zhang L, Poole K (1998) Role of the Multidrug Efflux Systems of *Pseudomonas aeruginosa* in Organic Solvent Tolerance. J Bacteriol 180:2987–2991. https://doi.org/10.1128/JB.180.11.2987-2991.1998

Persat A, Inclan YF, Engel JN, et al (2015) Type IV pili mechanochemically regulate virulence factors in *Pseudomonas aeruginosa*. Proc Natl Acad Sci USA 112:7563–7568. https://doi.org/10.1073/pnas.1502025112

Van Hoek AHAM, Mevius D, Guerra B, et al (2011) Acquired Antibiotic Resistance Genes: An Overview. Front Microbio 2:. https://doi.org/10.3389/fmicb.2011.00203
